# 2498: Individual patient outcome predictions using supervised learning methods

**DOI:** 10.1017/cts.2017.81

**Published:** 2018-05-10

**Authors:** Abiel Roche-Lima, Patricia Ordoñez, Nelson Schwarz, Adnel Figueroa-Jiménez, Leonardo A. Garcia-Lebron

**Affiliations:** University of Puerto Rico-Medical Sciences Campus, San Juan, Puerto Rico

## Abstract

OBJECTIVES/SPECIFIC AIMS: To learn the edit distance costs of a symbolic univariate time series representation through a stochastic finite-state transducer to predict patient outcomes in intensive care units. METHODS/STUDY POPULATION: High frequency data of patients in intensive care units were used as a data set. The nearest neighbor method with edit distance costs (learned by the FST) were used to classify the patient status within an hour after 10 hours of data. Several experiments were developed to estimate the parameters that better fit the model regarding the prediction metrics. RESULTS/ANTICIPATED RESULTS: Different metrics were obtained for the several parameters. These metrics were metrics (ie, accuracy, precision, and F-measure). DISCUSSION/SIGNIFICANCE OF IMPACT: Our best results are compared with published works, where most of the metrics (ie, accuracy, precision, and F-measure) were improved.

